# Meteorological Register

**Published:** 1814-04

**Authors:** C. Blunt

**Affiliations:** Philosophical Instrument Maker, No. 38, Tavistock-Street, Covent-Garden


### METEOROLOGICAL REGISTER.

*From February 25th, to March 25th,* 1814.

Kept by C. BLUNT, Philosophical Instrument Maker, No. 38, Tavistock-Street, Covent-Garden.

| Moon. | Day. | Wind. | Barometrical Pressure. | | Temperature. | | |
|---|---|---|---|---|---|---|---|
| | | | Max. | Min. | Max. | Min. | |
| | 26 | SE | 30·05 | 29·95 | 30 | 23 | Fair |
| | 27 | SW | 30.08 | 30·— | 30 | 26 | Fair |
| ☽ | 28 | SW | 29·83 | 29·30 | 40 | 35 | Fair |
| | 1 | W | 29·05 | 28·86 | 52 | 33 | Rain |
| | 2 | W | 28·90 | 28·79 | 39 | 29 | Rain |
| | 3 | NE | 29·10 | 28·90 | 40 | 30 | Rain |
| | 4 | NE | 29·40 | 29·18 | 36 | 28 | Snow |
| | 5 | E | 29·80 | 29·52 | 37 | 28 | Snow |
| | 6 | SE | 29·81 | 29·70 | 36 | 29 | Snow |
| ○ | 7 | SE | 29·79 | 29·65 | 33 | 23 | Snow |
| | 8 | E | 29·79 | 29·70 | 36 | 25 | Snow |
| | 9 | E by S | 29·64 | 29·56 | 33 | 28 | Snow & Sleet |
| | 10 | SE | 29 52 | 29 47 | 33 | 29 | Snow |
| | 11 | E | 29·60 | 29·45 | 34 | 27 | Snow |
| | 12 | NE | 29 77 | 29·69 | 35 | 28 | Cloudy |
| | 13 | NE | 30·— | 29.78 | 29 | 26 | Cloudy |
| ☾ | 14 | E | 30·26 | 30·18 | 35 | 24 | Cloudy |
| | 15 | E | 30·38 | 30·30 | 34 | 26 | Cloudy |
| | 16 | NE | 30 36 | 30·31 | 35 | 27 | Cloudy |
| | 17 | NE | 30·30 | 30·25 | 30 | 26 | Cloudy |
| | 18 | NE | 30·21 | 30 01 | 33 | 27 | Cloudy |
| | 19 | E | 29·96 | 29·82 | 35 | 29 | Cloudy |
| | 20 | E | 29 80 | 29·61 | 49 | 28 | Fair |
| ● | 21 | SE | 29 65 | 29.62 | 49 | 26 | Rain |
| | 22 | S | 29 69 | 29·66 | 48 | 36 | Fair |
| | 23 | S by W | 29·79 | 29·76 | 51 | 36 | Fair |
| | 24 | W | 29·72 | 29·64 | 50 | 42 | Wet |
| | 25 | W | 29·65 | 29 64 | 50 | 42 | Cloudy |

RESULTS.

| | |
|---|---|
| Mean barometrical pressure 29·71 | Mean temperature 33·7 deg. |
| Maximum 30·38 inc. the wind at E | Maximum 52, the wind at W |
| Minimum 28 90 ——— W | Minimum 23, ——— SE |

*Scale exhibiting the prevailing Winds during the Month.*

| N | NE | E | SE | S | SW | W | NW |
|---|---|---|---|---|---|---|---|
| 0 | 7 | 7 | 6 | 1 | 3 | 4 | 0 |

From the 26th ult. to the 4th inst. mean temperature 34·3

4th inst. —— 11th inst. ——————— 30·9

11th inst. —— 18th inst. ——————— 29·9

18th inst. —— 25th inst. ——————— 40·2

March 26th.—From the 20th inst. we may date the termination of a frost, of which, considering both duration and intensity, we have but few instances in this country. That of 1794 was more severe at intervals, but was not uniformly so: its duration was less by many weeks. That of 1789, when the Thames was passable to foot-passengers at Wapping, was of more uniform severity, but lasted only six weeks.

The

The mean temperature of each week of the past month, exhibits a gradual increase of temperature towards the close of the frost (taking the winds of the respective periods into the consideration.)

It is not unworthy of notice, that what must be considered the breaking up of the frost, took place on the day of the vernal equinox, and not many hours preceding the change of the moon.

On the 20th, about noon, the thermometer rose from 35 to 49 within two hours; and it was not a little extraordinary at this time, (the thermometer at 49 in the shade) and with a bright unclouded sun smiling over them, to witness at least one hundred persons skaiting on the middle of the Serpentine river, and seeming entirely unconscious either of the rapid changes going on around them, or of their own consequent danger.

The privations incident to a continued frost have greatly subsided with this felicitous change of weather; the labours of agriculture re-commence; the invalid crawls forth, hailing the dawn of spring, as his deliverance; and every man seems to expect its return with a quicker sense of its beauties and advantages.

With the next month, the results of a rain and evaporation gauge, and of an insulated electrical conductor, will be added to the present register.

Whilst the frost and cold weather continued, the diseases which prevailed were very similar to those of the preceding month. In some cases, catarrh has been accompanied with soreness of throat, and more fever than usual. Hooping-cough is frequent; measles rather rare. In Westminster, very little fever has been observed; but numbers of people of all ages and conditions have perished, in whom the state of the weather appeared to have considerably influenced the fatal termination of the complaint, especially when it was an affection of the pulmonary organs. Bronchitis asthenica has carried off a great many middle-aged and elderly persons. In this complaint, bleeding is often very injurious.

